# Thoracotomy is better than thoracoscopic lobectomy in the lymph node dissection of lung cancer: a systematic review and meta-analysis

**DOI:** 10.1186/s12957-016-1038-7

**Published:** 2016-11-17

**Authors:** Wenxiong Zhang, Yiping Wei, Han Jiang, Jianjun Xu, Dongliang Yu

**Affiliations:** Department of Cardiothoracic surgery, The second affiliated hospital of Nanchang University, 1 Minde Rd, Nanchang, Jiangxi Province 330006 China

**Keywords:** Video-assisted thoracic surgery, Thoracotomy, Meta-analysis, Lung cancer, Lymph node dissection

## Abstract

**Background:**

The aim of this study was to investigate which surgical method is better in lymph node (LN) dissection of lung cancer.

**Methods:**

A comprehensive search of PubMed, Ovid MEDLINE, EMBASE, Web of Science, ScienceDirect, the Cochrane Library, Scopus, and Google Scholar was performed to identify studies comparing thoracoscopic lobectomy (video-assisted thoracic surgery (VATS) group) and thoracotomy (open group) in LN dissection.

**Results:**

Twenty-nine articles met the inclusion criteria and involved 2763 patients in the VATS group and 3484 patients in the open group. The meta-analysis showed that fewer total LNs (95% confidence interval [CI] −1.52 to −0.73, *p* < 0.0001) and N2 LNs (95% CI −1.25 to −0.10, *p* = 0.02) were dissected in the VATS group. A similar number of total LN stations, N2 LN stations, and N1 LNs were harvested in both groups. Only one study reported that fewer N1 LN stations were dissected in the VATS group (1.4 ± 0.5 vs. 1.6 ± 0.6, *p* = 0.04).

**Conclusions:**

Open lobectomy could achieve better LN dissection efficacy than thoracoscopic lobectomy in the treatment of lung cancer, especially in the N2 LNs dissection. These findings require validation by high-quality, large-scale randomized controlled trials.

## Background

Lung cancer is the leading cause of cancer deaths in many countries [1, 2]. Surgical treatment is the preferred treatment for early-stage non-small cell lung cancer (NSCLC). D’Cunha’s research showed that N1 and N2 lymph nodes (LNs) were positive in 27.5% of patients with lung cancer under lobectomy [3]. However, non-invasive examinations, such as computed tomography (CT) and positron emission tomography-computed tomography (PET-CT), are not sensitive and specific for the clinical staging of lung cancer. Video-assisted thoracic surgery (VATS) is the preferred surgical procedure, with fewer incidences of postoperative complications and a higher survival rate compared with thoracotomy [4–7]. However, whether VATS can achieve the same LN dissection efficacy is controversial, and there remains a lack of high-quality, large-scale clinical research.

To determine whether VATS can achieve the same LN dissection efficacy as thoracotomy in lung cancer, we performed a systemic review and meta-analysis.

## Methods

### Search strategy

MEDLINE and manual searches were performed by two investigators independently and in duplicate to identify all relevant scientific articles published from January 1990 to May 2016. The MEDLINE search was performed using PubMed, Ovid MEDLINE, EMBASE, Web of Science, ScienceDirect, The Cochrane Library, Scopus, and Google Scholar. The MeSH terms “lung cancer or lung neoplasm”, “thoracotomy or open surgery”, and “video-assisted thoracic surgery or VATS” and comparative study were used.

### Inclusion criteria and exclusion criteria

The following inclusion criteria were applied: (1) published in English, (2) compared the LN dissection of thoracoscopic lobectomy with thoracotomy in treating patients with lung cancer, and (3) the most recent study was chosen when duplication of data is in more than one article.

Reviews without original data, case reports, meta-analyses, letters, expert opinions, and animal studies were excluded. Studies on robotic-assisted VATS were also excluded.

### Data extraction

Two investigators independently extracted data from the eligible studies. The extracted data included first author, year of publication, geographical area, study design, duration of enrollment, information on preoperative staging, number of patients per group, LN number (LNN), and LN station number (LNS).

### Quality assessment for included studies

Two investigators independently assessed the quality of each included study using the Newcastle-Ottawa Scale (NOS) for non-randomized studies and the Jadad scale for randomized controlled trials (RCTs).

The NOS evaluates the quality of studies by analyzing three items: selection, comparability, and exposure. The scale assigns a maximum of nine points to each study: a maximum of four points for selection, two points for comparability, and three points for exposure. Therefore, the highest quality study would score nine points. In our analysis, high-quality studies were defined as those that scored nine or eight points; medium-quality studies were those that scored seven or six points [8].

The Jadad scale (five points) contained questions for three main parts: randomization, masking, and accountability of all patients (withdrawals and dropouts). Studies scored ≥3 points were considered as high quality [9].

### Statistical analysis

Meta-analysis was conducted by Review Manager 5.3 and SPSS 18.0, *p* value < 0.05 suggested statistically significant. The differences were compared between the two groups using analysis of variance for continuous variables and pooled relative risk (RR) with 95% confidence interval (CI) for categorical variables. We used *I* ^2^ and Cochran Q to evaluate the between-study heterogeneity. A random-effects model was adopted when the heterogeneity was significant (*p* ≤ 0.10 and *I* ^2^ > 50%); otherwise, a fixed-effects model was used. Rank correlation test of funnel plot asymmetry was used to assess the potential publication bias.

## Results

### Search results and quality assessment of the included studies

**Fig. 1:**
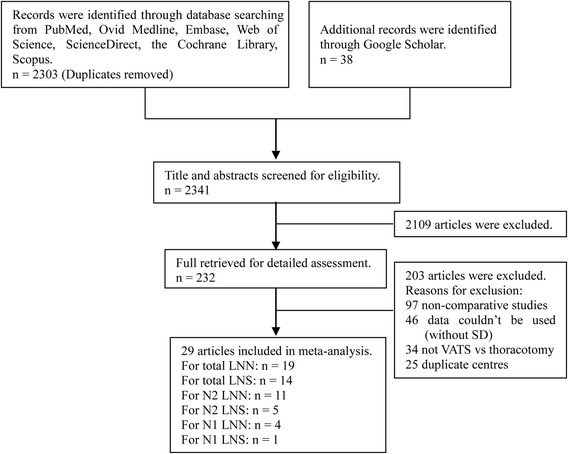
Flow diagram of screened and included papers

We initially identified 2341 publications from the database and reference list searches and reviewed 29 articles for final analysis (Fig. 1). The articles involved a total of 6247 patients, of whom 2763 underwent VATS and 3484 underwent thoracotomy. Of these 29 publications, three studies were RCTs and 26 were retrospective studies. According to the NOS and Jadad scales assessment scores, 23 articles were of good quality and the remaining six were medium quality. The baseline characteristics of these articles are listed in Table 1.

**Table 1:** Summary of the 29 trials included in the present meta-analysis

Study		Institution	Enrolled year		No. of patients		Clinical stage	Outcomes	Design	Quality
			VATS	Open	VATS	Open				
1995	Kirby [28] USA	Single	1991.10–1993.12	1991.10–1993.12	25	30	I	①	RCT	3
1998	Morikawa [29] JPN	Single	1996.04–1996.12	1995.01–1996.03	39	41	I–II	③	Retrospective	9
2000	Luketich [30] USA	Single	Not mentioned	Not mentioned	31	31	I	①	Retrospective	7
2000	Sugi [31] JPN	Single	1993.01–1994.06	1993.01–1994.06	48	52	Ia	③, ⑤	RCT	3
2001	Nomori [32] JPN	Single	1999.08–2000.12	1998.04–1999.07	33	33	I	①,⑦,⑧	Retrospective	7
2005	Watanabe [33] JPN	Single	1997–2004	1997–2004	221	190	I	①, ③, ⑦, ⑧	Retrospective	8
2006	Petersen [34] USA	Single	2001–2005	1996–2005	12	85	I–IV	②	Retrospective	8
2006	Shigemura [35] JPN	Multi	1999.01–2004.01	1999.01–2004.01	50	55	Ia	①	Retrospective	9
2008	Shiraishi [36] JPN	Single	1994.11–2005.10	1994.11–2005.10	20	55	I	③, ⑤	Retrospective	8
2008	Watanabe [37] JPN	Single	1997–2006	1997–2006	37	32	I	①, ②, ③, ④	Retrospective	8
2008	Whitson [38] USA	Single	Not mentioned	Not mentioned	6	7	Not mentioned	①	Retrospective	6
2009	Nakanishi [39] JPN	Single	2000.04–2007.01	2000.04–2007.01	13	14	I–IV	③	Retrospective	8
2009	Okur [40] TR	Single	2007.01–2007.11	2007.01–2007.11	20	28	I	②	Retrospective	8
2010	Denlinger [21] USA	Single	2000.01–2008.08	2000.01–2008.08	79	464	I	①, ③, ⑤	Retrospective	8
2011	D’Amico [41] USA	Single	2007.01–2010.09	2007.01–2010.09	199	189	I–III	②,④	Retrospective	7
2012	Bu [42] CHN	Single	2001.05–2011.04	2001.05–2011.04	46	87	Not mentioned	①, ②	Retrospective	7
2012	Li [43] CHN	Single	2006.09–2009.12	2006.09–2009.12	29	47	I	③, ④	Retrospective	8
2012	Licht [44] DNK	Multi	2007.01–2011.12	2007.01–2011.12	717	796	I	②	Retrospective	8
2013	Fan [45] CHN	Single	2005.01–2010.12	2005.01–2010.12	79	77	I–II	①, ②	Retrospective	8
2013	Lee [22] USA	Single	1990.05–2011.12	1990.05–2011.12	141	115	Not mentioned	①, ②, ③, ④, ⑤, ⑥	Retrospective	8
2013	Palade [46] GER	Single	2008.05–2011.12	2008.05–2011.12	32	32	I	①, ⑦, ⑧	RCT	3
2013	Zhong [47] CHN	Single	2006.03–2011.08	2006.03–2011.08	67	90	I	①, ②, ③, ④	Retrospective	8
2014	Li [48] CHN	Single	2011.02–2013.02	2011.02–2013.02	21	32	I–II	①	Retrospective	8
2014	Stephens [4] USA	Single	2002.01–2011.12	2002.01–2011.12	307	307	I	②	Retrospective	8
2015	Cai [49] CHN	Single	2010.01–2012.05	2010.01–2012.05	71	67	I–II	①, ②	Retrospective	9
2015	Kuritzky [50] USA	Single	2007–2012	2007–2012	74	224	I	②	Retrospective	8
2015	Murakawa [51] JPN	Single	2001–2010	2001–2010	101	101	I	①, ②	Retrospective	8
2015	Nwogu [6] USA	Multi	2004.10–2010.06	2004.10–2010.06	175	175	I–II	①, ②	Retrospective	7
2015	Zhang [52] CHN	Single	2012.10–2013.11	2012.10–2013.11	70	28	I	①	Retrospective	9

① total lymph node number, ② total lymph node station number, ③ N2 LNN, ④ N2 LNS, ⑤ N1 LNN, ⑥ N1 LNS, ⑦ left-side LNN, ⑧ right-side LNN

*CHN* China, *DNK* Denmark, *JPN* Japan, *GER* Germany, *TR* Turkey, *USA* United States of America, *RCT* randomized controlled trial

### Comparison of total LNN and LNS

**Fig. 2:**
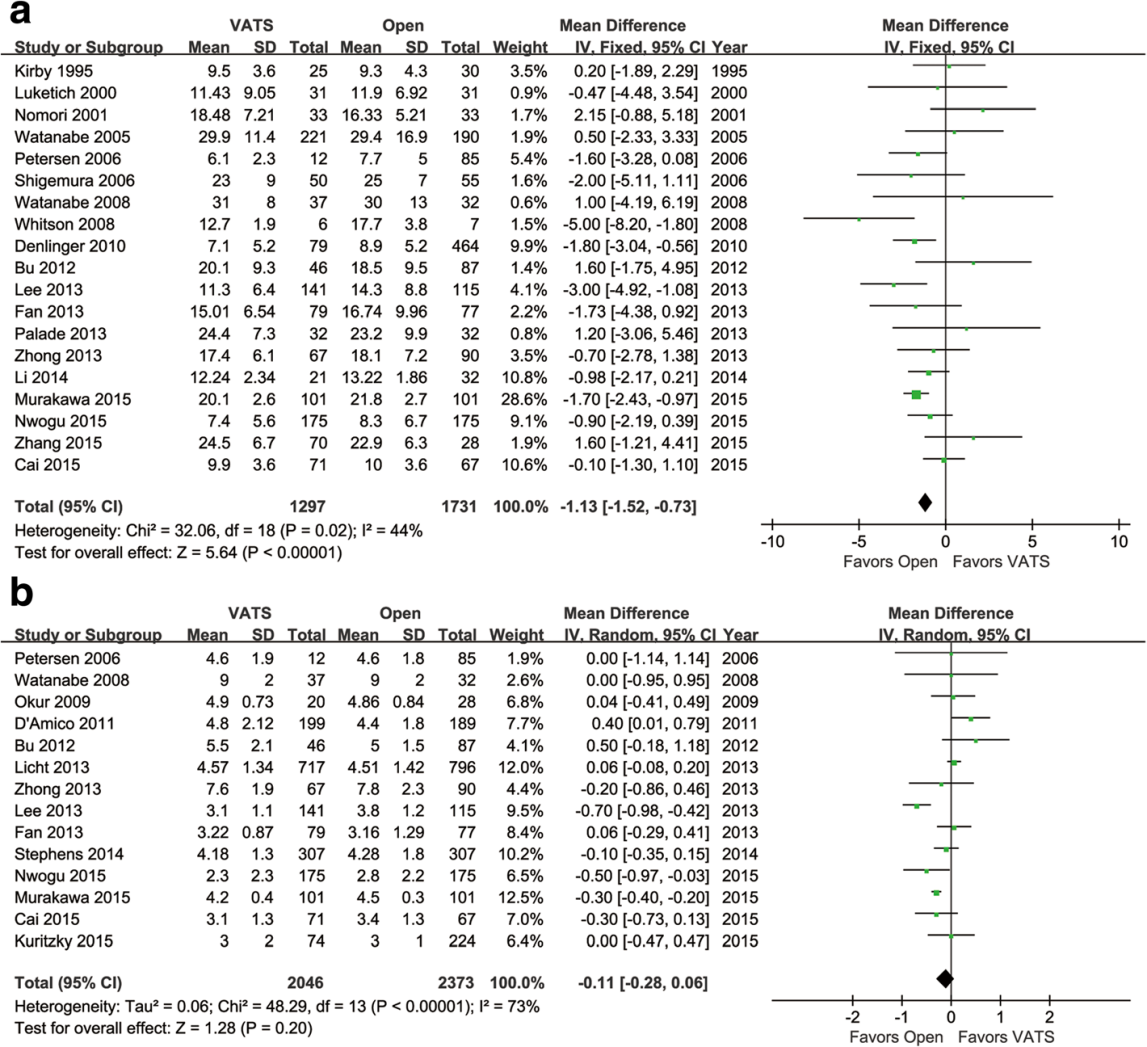
Forest plot of the mean difference in total LNN (**a**) and LNS (**b**) in the VATS group vs. the open group

We identified 19 articles for total LNN comparison. They involved 1297 patients in the VATS group and 1731 patients in the open group (thoracotomy). The heterogeneity between these studies was acceptable (*p* = 0.02, *I* ^2^ = 44%). Fewer total LNs were dissected in the VATS group as compared with the open group (95% CI −1.52 to −0.73, *p* < 0.00001, Fig. 2a).

Fourteen articles were identified for total LNS comparison. They involved 2046 patients in the VATS group and 2373 patients in the open group. The mean difference in total LNS between the two groups was not significant (95% CI −0.28 to 0.06, *p* = 0.20), with significant heterogeneity across studies (*p* < 0.00001, *I* ^2^ = 73%, Fig. 2b).

### Comparison of N2 LNN and LNS

**Fig. 3:**
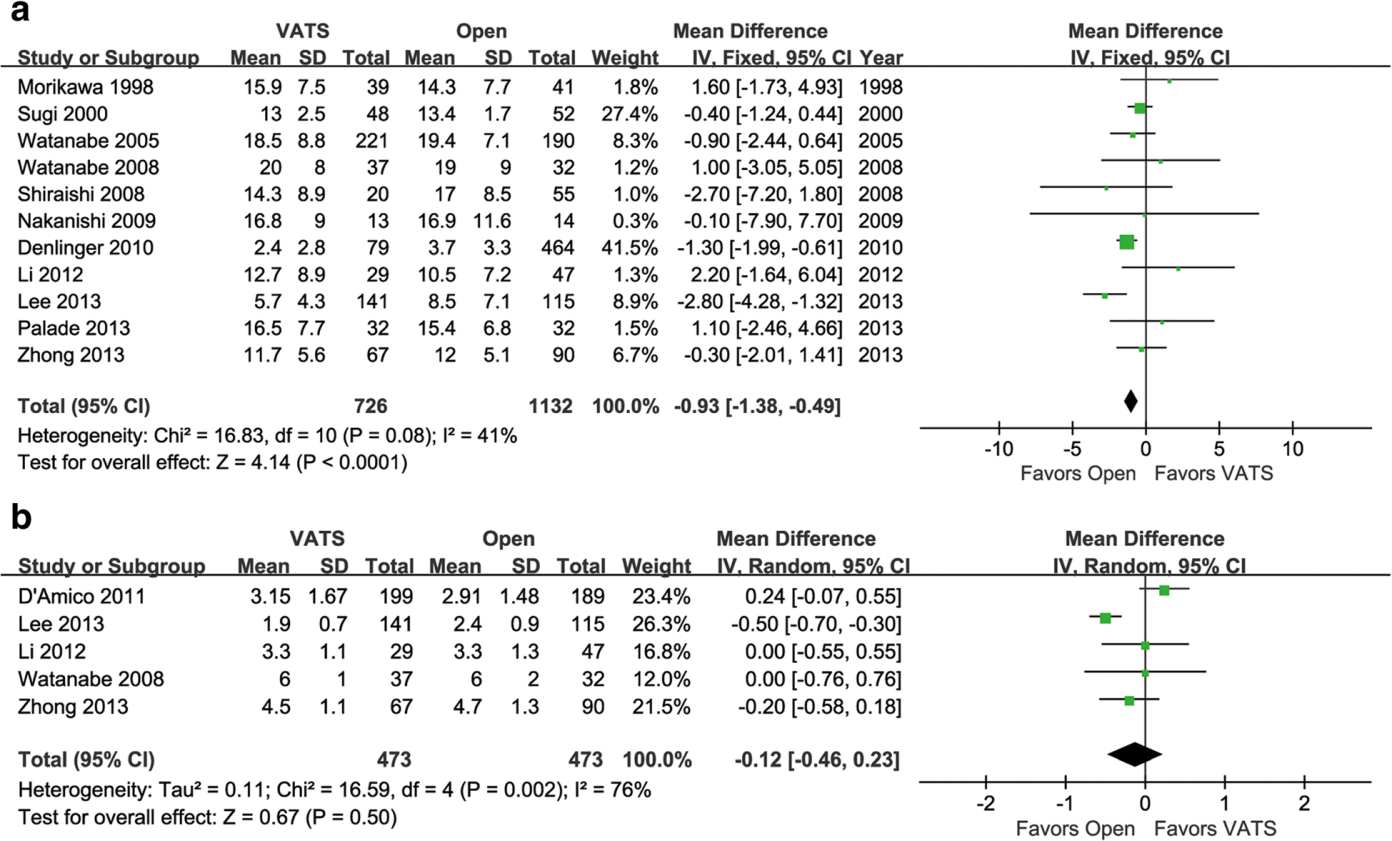
Forest plot of the mean difference in N2 LNN (**a**) and LNS (**b**) in the VATS group vs. the open group

Eleven articles were identified for N2 LNN comparison. They involved 726 patients in the VATS group and 1132 patients in the open group. The heterogeneity between these studies was acceptable (*p* = 0.08, *I* ^2^ = 41%). Fewer N2 LNs were dissected in the VATS group as compared with the open group (95% CI −1.38 to −0.49, *p* < 0.0001, Fig. 3a).

Five articles were identified for N2 LNS comparison. They involved 473 patients in the VATS group and 473 patients in the open group. The mean difference in N2 LNS between the two groups was not significant (95% CI −0.46 to 0.23, *p* = 0.50), with significant heterogeneity across studies (*p* = 0.002, *I* ^2^ = 76%, Fig. 3b).

### Comparison of N1 LNN and LNS

**Fig. 4:**
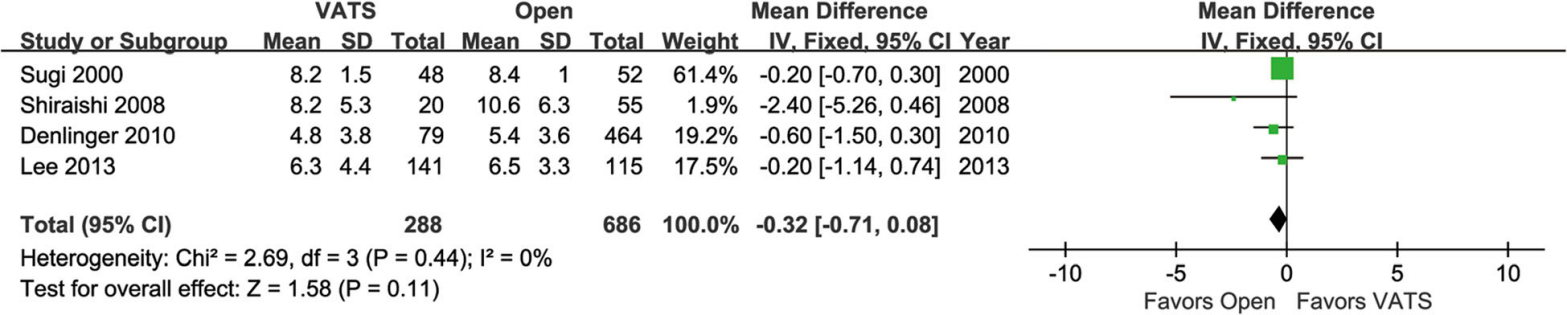
Forest plot of the mean difference in N1 LNN in the VATS group vs. the open group

Four articles were identified for N1 LNN comparison. They involved 288 patients in the VATS group and 686 patients in the open group. The heterogeneity between these studies was acceptable (*p* = 0.44, *I* ^2^ = 60%). The mean difference in N1 LNN between the two groups was not significant (95% CI −0.71 to 0.08, *p* = 0.11, Fig. 4).

Only one article was identified for N1 LNS comparison. They involved 141 patients in the VATS group and 115 patients in the open group. The result showed that fewer N1 LN stations were dissected in the VATS group (1.4 ± 0.5 vs. 1.6 ± 0.6, *p* = 0.04).

### Publication bias

**Fig. 5:**
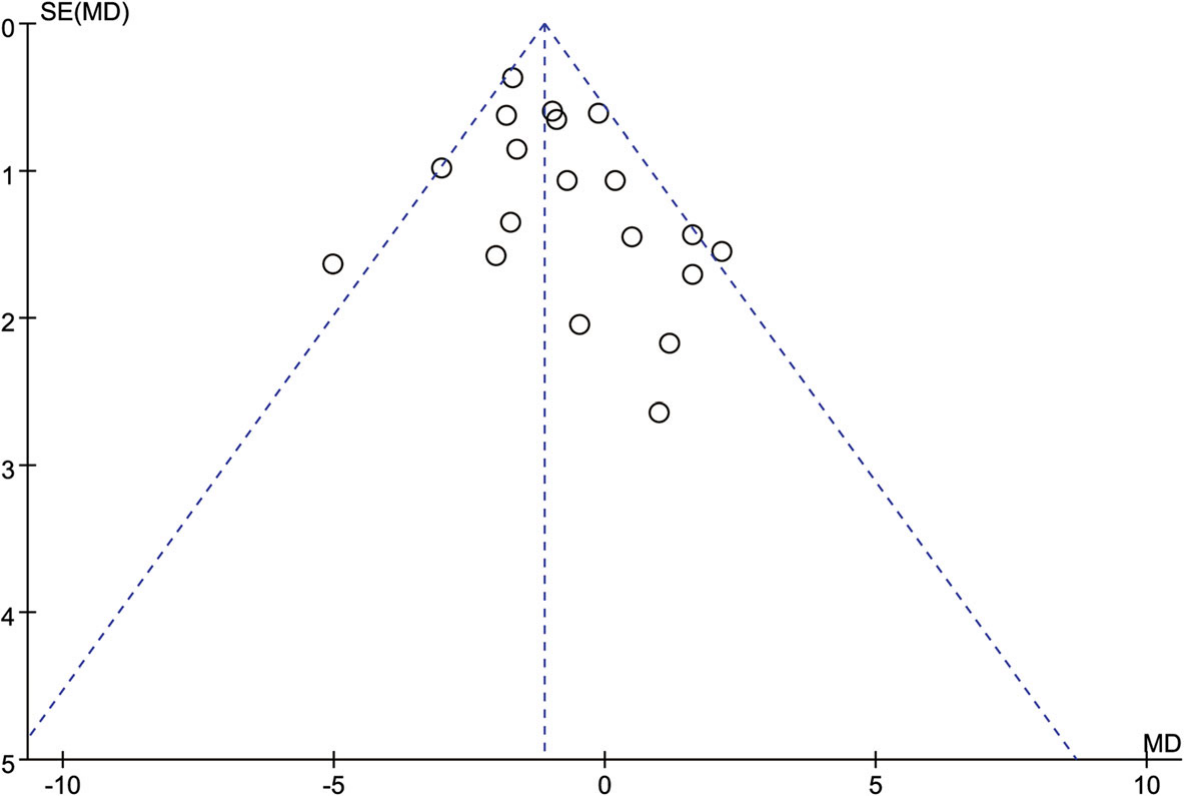
Funnel plot of the mean difference in total LNN in the VATS group vs. the open group

The funnel plot for publication bias (standard error by total LNN comparison) demonstrated marked evidence of symmetry (Fig. 5), indicating no publication bias. The combined effect size yielded a *Z* value of 5.64, with a corresponding *p* < 0.00001. This result indicates that the fail-safe *N* value was relevant.

## Discussion

The presence or absence of mediastinal LN metastases is a critical component to accurate staging and therefore a key component of the surgical management of NSCLC [10]. Both overall and disease-free survival have been associated with the number of LNs dissected [11, 12]. Lardinois compared LNs dissection versus sampling; the results showed a longer disease-free survival and better local tumor control in dissection groups [13]. Focusing on stage IA lung cancer, Xu reported a similar result and suggested that the number of N2 stations served as a more significant prognostic factor [14]. As a result, current guidelines from the National Comprehensive Cancer Network and European Society of Thoracic Surgeons recommend that all patients with resectable NSCLC should have a complete systematic nodal dissection, with at least three N2 stations dissected [15, 16].

Although VATS is associated with many benefits in comparison to thoracotomy, whether VATS can achieve the same LN dissection efficacy in patients with lung cancer remains controversial [7, 17, 18]. Flores reported that the surgical field in VATS facilitated the dissection of LNs adjacent to the blood vessels and the trachea and found that smaller nodes achieved better LN dissection [19]. Ramos showed that VATS dissected more total LNS (5.1 ± 1.1 vs. 4.5 ± 1.2, *p* < 0.001) and mediastinal LNS (3.4 ± 0.9 vs. 3.2 ± 0.9, *p* = 0.022) when compared with thoracotomy [20]. By contrast, Denlinger reported that significantly more overall LNs were dissected in the open group than in the VATS group (8.9 ± 5.2 vs. 7.1 ± 5.2, *p* = 0.0006) [21]. Similar results were reported by Lee and colleagues in their retrospective study of LN evaluation achieved by VATS lobectomy compared with that by open lobectomy [22]. A secondary analysis including 752 original participants of the American College of Surgeons Oncology Group Z0030 trial compared patients who underwent lobectomy by VATS with patients who underwent thoracotomy. The results showed that there was no significant difference in the overall number of LNs retrieved between the two groups (15 vs. 19, *p* = 0.147) [23].

The present study included a total of 7568 patients from nine countries, providing the most comprehensive evidence for LN dissection efficacy by VATS to date. The meta-analysis showed that fewer total LNN and similar total LNS were dissected in the VATS group as compared with that in the open group. If we only included studies focusing on clinical stage I lung cancer, the results were similar (total LNN, 95% CI −1.67 to −0.61, *p* < 0.0001; total LNS, 95% CI −0.62 to 0.02, *p* = 0.07). It suggests that surgeons may not have the ability to perform systematic lymphadenectomy in VATS or ignore the importance of systematic lymphadenectomy for various reasons (earlier tumor stage, worrying about damage to vital organs, and so on).

In the comparison of N1 and N2 LN dissection, our results showed that similar number of N1 LNN and N2 LNS could be harvested by VATS, while fewer N2 LNN were harvested by VATS as compared with thoracotomy. Only one article reported on N1 LNS comparison between the two groups and showed better efficiency in the open group (1.4 ± 0.5 vs. 1.6 ± 0.6, *p* = 0.04) [22].

It was controversial that removing more N2 LNs could increase the accuracy of clinical staging of NSCLC. Boffa et al. compared the completeness of surgical LN evaluation during anatomic resection of primary lung cancer by open and VATS approaches in 11,531 patients from The Society of Thoracic Surgeons-General Thoracic Database. The results showed nodal upstaging in 14.3% (1024 patients) of the open group and in 11.6% (508 patients) of the VATS group (*p* < 0.001). The study suggested that surgeons should be encouraged to apply a systematic approach to hilar and peribronchial LN dissection during VATS lobectomy for lung cancer, particularly as they were adopting this approach [24].

Some surgeons might also worry about the complications caused by the systematic LN dissection. As with open thoracotomy, systematic mediastinal LN dissection under VATS may increase the risk of intraoperative bleeding (bronchial arteries, etc.), tracheobronchial injury, recurrent nerve injury, prolonged air leak, atrial fibrillation, and pulmonary edema [25]. In other papers, Watanabe et al. reported similar mortality and morbidity of mediastinal LN dissection by VATS vs. open lobectomy, indicating that systematic mediastinal LN dissection by VATS is a safe procedure [26]. Zhang et al. compared complications such as chylothorax and nerve injury between VATS and open thoracotomy in a meta-analysis. The results showed that these events were similar in both groups [27].

The possible limitations of our study must be considered when interpreting the findings described herein. First, including only English papers might have resulted in language bias. Second, including 7568 participants from 36 studies with only three RCTs might have weakened the quality of the results. Third, the number of dissected LNs varied significantly between the included studies. Different doctors have different understanding of LN dissection and might be at different stages of the learning curve. Some data did not meet the National Comprehensive Cancer Network guide requirements for lung cancer surgery treatment of systematic LN dissection, which may have affected the reliability of the results. Fourth, there is great potential for LN fragmentation during dissection. Different pathologists and different counting procedures might lead to false LNN counts, which might increase the heterogeneity between studies but would not alter the overall results. Finally, we did not analyze the survival difference between VATS and open thoracotomy. Our analysis compared LN harvest capability between two evaluation procedures only from a surgical point of view and tried to give further proof of satisfied oncologic efficacy by VATS.

## Conclusions

Less total and mediastinal LNs were evaluated with VATS than with thoracotomy in the present study. Both approaches harvested a similar number of total LN stations, mediastinal LN stations, and N1 LNs. However, owing to the possible existing bias in the original studies, inter-study heterogeneity, and the inherent limitations of our meta-analysis, the findings require validation in high-quality, large-scale RCTs.

## Abbreviations

CHN: China
CI: Confidence interval
CT: Computed tomography
DNK: Denmark
GER: Germany
JPN: Japan
LN: Lymph node
LNN: Lymph node number
LNS: LN station number
NOS: Newcastle-Ottawa Scale
NSCLC: Non-small cell lung cancer
PET: Positron emission tomography
RCTs: Randomized controlled trials
RR: Relative risk
TR: Turkey
USA: United States of America
VATS: Video-assisted thoracic surgery
